# Corona Discharge Characteristics under Variable Frequency and Pressure Environments

**DOI:** 10.3390/s21196676

**Published:** 2021-10-08

**Authors:** Pau Bas-Calopa, Jordi-Roger Riba, Manuel Moreno-Eguilaz

**Affiliations:** 1 Electrical Engineering Department, Universitat Politècnica de Catalunya, 08222 Terrassa, Spain; pau.bas@upc.edu; 2 Electronics Engineering Department, Universitat Politècnica de Catalunya, 08222 Terrassa, Spain; manuel.moreno.eguilaz@upc.edu

**Keywords:** more electric aircraft, electrical discharges, visual corona, corona extinction voltage, variable frequency, low pressure

## Abstract

More electric aircrafts (MEAs) are paving the path to all electric aircrafts (AEAs), which make a much more intensive use of electrical power than conventional aircrafts. Due to the strict weight requirements, both MEA and AEA systems require to increase the distribution voltage in order to limit the required electrical current. Under this paradigm new issues arise, in part due to the voltage rise and in part because of the harsh environments found in aircrafts systems, especially those related to low pressure and high-electric frequency operation. Increased voltage levels, high-operating frequencies, low-pressure environments and reduced distances between wires pose insulation systems at risk, so partial discharges (PDs) and electrical breakdown are more likely to occur. This paper performs an experimental analysis of the effect of low-pressure environments and high-operating frequencies on the visual corona voltage, since corona discharges occurrence is directly related to arc tracking and insulation degradation in wiring systems. To this end, a rod-to-plane electrode configuration is tested in the 20–100 kPa and 50–1000 Hz ranges, these ranges cover most aircraft applications, so that the corona extinction voltage is experimentally determined by using a low-cost high-resolution CMOS imaging sensor which is sensitive to the visible and near ultraviolet (UV) spectra. The imaging sensor locates the discharge points and the intensity of the discharge, offering simplicity and low-cost measurements with high sensitivity. Moreover, to assess the performance of such sensor, the discharges are also acquired by analyzing the leakage current using an inexpensive resistor and a fast oscilloscope. The experimental data presented in this paper can be useful in designing insulation systems for MEA and AEA applications.

## 1. Introduction

More electric aircrafts (MEAs) allow for reducing weight [1], fuel consumption, greenhouse gas emissions, operation and maintenance costs and boosting overall system efficiency when compared with conventional aircrafts [2]. However, engineers are facing important challenges due to the increased voltage levels MEAs require, the increase in the power density and the d*v*/d*t*, or the reduction in distances between electrical wires, thus increase the likelihood of electric arc occurrence [3,4] with the consequent safety risks.

Jet aircrafts typically fly at altitudes between 33,000 and 42,000 feet (10,000 m to 12,800 m) [5], thus operating under harsh environmental conditions. Some electric and electronic aircraft systems operate in unpressurized zones [6], so electric and electronic aircraft systems must be designed to operate under a broad range of pressures, in the range 1 atm to 0.15 atm [7].

The development of MEA and AEA systems is accompanied by a rise of the distribution voltage levels, since for a given power, the lower the current, the higher the voltage, and vice versa. However, according to Paschen’s law, when operating at higher voltage levels and reduced pressure, there is risk of partial discharges (PDs) and electric breakdown [8], the inception voltages of such discharges being below the ones found at sea level [9,10].

Direct current (dc) distribution systems of current aircrafts are operated at 28 V, 270 V (±135 V) or 540 V (±270 V), whereas alternating current (ac) distribution systems are operated at 230 V or 115 V phase voltage with variable or wide frequency (typically 320–800 Hz) [11], or 230 V or 115 V phase voltage with constant frequency (400 Hz) [12,13]. In AEA, voltage levels in the range 2 to 3 kV seem advantageous [13]. It is believed that ac distribution systems in the voltage range between 0.6 and 2 kV lead to wiring systems with less weight, reduced power losses and higher efficiency. However, above 2 kV, additional insulation requirements add extra mass to the system, thus needing careful analysis [13]. Because of the need of more electrical power, next generations MEA aircrafts will probably raise the distribution voltage above 1 kV [14,15]. According to NASA, future aerospace systems can operate at voltages up to 20 kV (designed for 40 kV), with high-frequency operation (400 Hz to 4000 Hz) [16]. The combination of low pressure, high voltage and high-operating frequencies stresses insulation systems [13], with the consequent degradation risk due to partial discharge and arc tracking occurrence [17,18] because electrical discharge inception voltages can be much lower than those at sea level [1].

Wiring issues in aircrafts due to electrical discharges and arc tracking leading to insulation degradation have caused catastrophic accidents [13]. Different insulation materials have been proposed to combat insulation degradation [19,20]. This is of paramount importance because MEA and AEA make an increasing use of electric and electronic apparatus and devices, so polymer insulation materials are inevitably exposed to harsh and varying environments. Thus, care must be taken in selecting appropriate insulating materials since reliability is an issue [21]. Before electric breakdown occurrence, partial discharges (PDs) appear, PDs being discharges that do not entirely channel the insulation between two electrodes [22]. They are roughly classified as internal discharges, external discharges and corona discharges. Although short duration PDs are usually harmless, when they persist over time, they tend to generate important insulation damage in polymeric materials because PDs can produce a partially conductive path or track on the insulation outer surface, thus favoring the flow of an electric current and ultimately arc tracking activity or even complete electrical breakdown [23]. Arc tracking occurring in organic (polymeric) insulation systems, damages the polymer material, which shifts from insulating to conductor because of the tremendous thermal shocks due to the electron bombardment generated by the electrical discharge [24]. This effect also breaks the polymeric chains and degrades the insulation, generating conducting carbon tracks, which reduce the insulating properties of the polymer surface and promote electrical breakdown [25], fire hazard [26] and explosions [27], even at very low voltage [28]. Atmospheric pressure, applied voltage, supply frequency and geometry are dominant variables to determine corona discharge inception and extinction levels.

It is worth noting that reliability and safety are key points in aircraft systems. To design reliable aircraft insulation systems, it is necessary to have a deep knowledge of the conditions leading to a corona [6] as a function of environmental pressure and supply frequency, because if these conditions are not controlled, they can lead to damaging effects, including arc tracking and electrical breakdown [2]. To better understand the effect of low pressure and supply frequency on the development of electrical discharges, it is imperative to run extensive test plans. Due to the difficulty to operate under low-pressure environments and using high-voltage generators with adjustable frequency, there is a lack of experimental data obtained under conditions compatible with aeronautic environments. This paper aims to contribute in this field. In addition, some of the studies are focused to analyze the disruptive spark breakdown [29,30], but non-uniform gaps can lead to corona inception and extinction voltages much lower than those required to ignite disruptive or breakdown discharges.

To analyze the effect of low-pressure environments jointly with the effect of the supply frequency, a rod-to-plane electrode configuration is tested in the 20–100 kPa and 50–1000 Hz intervals, these ranges account for the wide range of pressure and frequencies found in aircraft applications.

The detection of partial discharges and arcing activity in aircrafts in the very early stage is a problem that remains unsolved, so there is an imperious need to develop sensor systems to solve this important safety problem. Although there are several sensors that potentially can be applied to detect electrical discharges such as PD detection, antennas to detect electromagnetic noise and radio interference voltage, or acoustic sensors, they are too complex or are severely affected by the noise found in aeronautic applications. In addition, these methods do not directly allow to locate the discharge points. Therefore, this paper focus on the visible-UV light emitted by the electrical discharges because this method offers immunity to noise, while allowing to locate the discharge points.

It is known that the corona effect generates visible (mainly blue) and ultraviolet (UV) light [31]. Thus, by using optical sensors sensitive to these spectral regions, it is possible to detect the corona discharges in the early stage [2]. A corona can also be detected by means of other methods, which are usually more complex, such as optical spectrophotometers [32], audible noise meters [33], PD and radio interference voltage (RIV) detectors [34] or UHF sensors [35]. However, the simpler and straightforward way to locate the discharge point is by using visible-UV imaging sensors. Therefore, to determine the conditions leading to a corona, the corona extinction voltage is determined by using a low-cost high-resolution CMOS imaging sensor. This sensor is sensitive to the visible and near ultraviolet spectral ranges, and the discharge points are identified from the images generated by the CMOS sensor, as well as the intensity of the discharge, thus offering high sensitivity, simplicity low-cost measurements and immunity to electromagnetic noise. Results attained with the imaging sensor are compared with those obtained by analyzing the leakage current. Experimental data presented in this paper can be useful to design insulation systems for future MEA and AEA applications, thus ensuring the reliability of aircraft insulation systems for electrical and electronic circuits.

Specific objectives of this research work include determining the combined effect of pressure and frequency on visual corona and specifically on corona extinction voltage (CEV) for aeronautics applications using a low-cost CMOS imaging sensor, and to compare the sensitivity of such sensor with that of a leakage current sensor.

The paper is organized as follows. Section 2 details the experimental setup to generate a variable frequency high voltage and the instrumentation used, as well as the sensors used to detect the corona extinction voltage. The experimental results are presented in Section 3, whereas Section 4 discusses the results attained. Finally, the conclusions of the paper are developed in Section 5.

## 2. Experimental Setup

**Figure 1:**
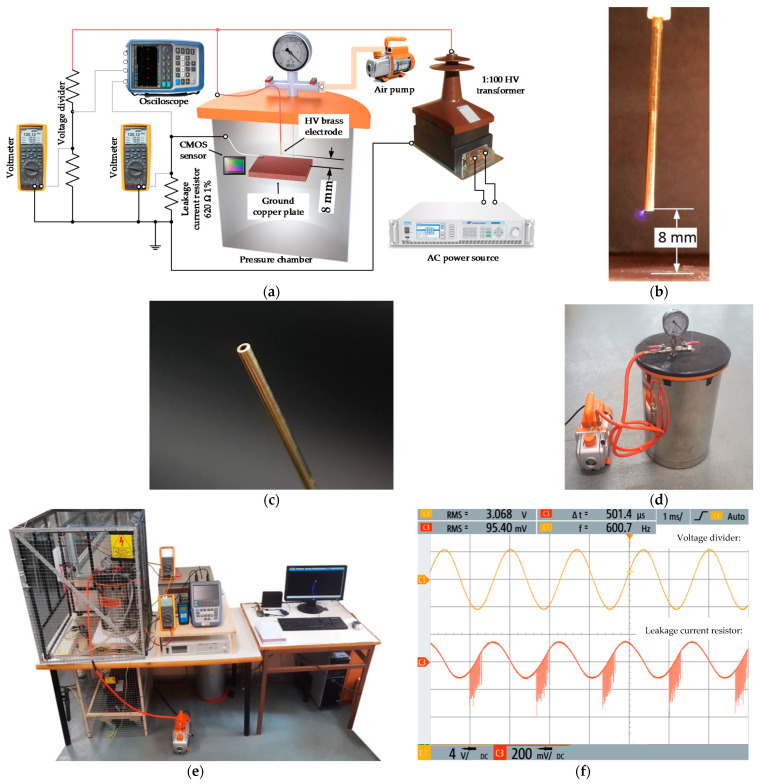
Experimental setup. (**a**) Sketch of the experimental setup and the instrumentation used in the high-voltage tests at variable pressure, frequency and voltage; (**b**) photograph of the rod-to-plane electrode used in the experiments; (**c**) detail of the tip of the electrode; (**d**) low-pressure chamber; (**e**) photograph of the experimental setup including the low-pressure chamber; (**f**) snapshot of the oscilloscope used to detect corona activity connected to the terminals of the leakage current resistor.

Corona experiments were performed inside a pressurized chamber that allows reducing the pressure from 100% to 20% of the pressure at sea level, i.e., from 100 kPa to 20 kPa approximately, covering the altitude/pressure interval of commercial jet liners. The low-pressure chamber is composed of a stainless-steel cylindrical container (diameter = 130 mm, height = 375 mm) with a sealed methacrylate lid to allow the wireless imaging sensor to transmit the long-exposure photographs to a computer placed outside the low-pressure chamber, as displayed in Figure 1c. The pressure is regulated using a vacuum pump (1/4 HP, 0.085 m^3^/min, Bacoeng BA-1, Bacoeng, Suzhou, China) and a manometer (76 mmHg, ±2.5%, Bacoeng, Suzhou, China). Experiments were conducted at a constant room temperature of 25 °C. The humidity effect was not studied but limited to below 25% during the experiments.

The applied voltage and supply frequency were regulated by means of a SP300VAC600W programmable ac source (600 W, 0–300 V, ±0.1 V, 15–1000 Hz, APM Technologies, Dongguan, China) following the IEC61000-4-14 standard. A high-voltage instrument transformer (single-phase, turns ratio 1:100, maximum voltage 36 kV, 600 VA, VKPE-36, Laboratorio Electrotécnico, Cornellà de Llobregat, Spain) was connected to the output of the SP300VAC600W programmable ac source to step up the output voltage provided by this source.

A voltage divider with a voltage ratio of 1000:1 was used to measure the high voltage at the output of the high-voltage transformer, so that the load voltage was measured with a calibrated true-RMS voltmeter (0–1000 V_RMS_, 0.4%, 0–10 A, Fluke 289, Fluke, Everett, WA, USA).

The rod-to-plane gap is composed of a MBT5M brass tube (Albion Alloys, Poole, UK) with outer and inner diameters of Ø = 1.5 mm and Ø = 0.8 mm, respectively. The tip of the electrode was placed at a height of 8 mm above a grounded flat copper plane. A rod-to-plane arrangement was used in this work because this geometry is among the reference gaps used in high-voltage applications [36], thus allowing the generation of PDs. The tip was cut with a hacksaw for metals and polished with a metal grinding wheel (fine grain 220 g, 2800 rev/min). This geometry was chosen in order to generate a corona before arc appearance under the conditions analyzed in this work (20–100 kPa, 50–1000 Hz, 25 °C, humidity < 25%, <6 kV) being compatible with the dimensions of the low-pressure chamber.

The experimental corona extinction voltage (CEV) values shown and analyzed in Section 3 are measured by the means of two detection methods. The first method is based on visual corona tests, a corona representing a pre-arc condition in its very early stage before obvious damage in the insulation can be appreciated. To detect the visual corona phenomenon and locate the discharge area, a high-resolution low-cost back-illuminated CMOS imaging sensor (sensor size 8.0 mm, cell size 0.8 μm × 0.8 μm, 8000 × 6000 pixels, 48 Mpixels, 30 frames/second, lens focal 17.9 mm, quad Bayer filter array, images in raw format, IMX586, Sony, Tokyo, Japan) was used, because back-illuminated CMOS sensors are sensitive to visible and UV light [37]. To increase the sensitivity of the measurements, long-exposure pictures were taken for 32s in manual focus mode, selecting an ISO of 400. This is a low-cost sensor that allows for locating corona discharge regions, as well as quantifying the intensity of the discharges, thus easing maintenance tasks. This sensor also enables reducing the costs and complexity of the instrumentation while offering excellent measurement sensitivity and accuracy. Due to a special arrangement of the photodiodes, back-illuminated CMOS imaging sensors allow capturing more light compared with conventional CMOS sensors, thus performing better under low-light conditions, particularly in the UV spectrum [37,38].

To determine the existence of a corona in the images taken by the CMOS sensor, they were first converted to grayscale (rgb2gray function in Matlab^®^). Next, the mean value of the pixels of a selected window centered near the corona focus was calculated and compared with the mean value of the pixels from the rest of the image. If the first value is greater than the second by 5%, it is assumed that there is a corona. This simple processing approach is quite immune to the effect of external light (partial darkness).

The second method is based on measuring the leakage current. In this case the sensing system consists of a 620 Ω ± 1% low-inductance resistor connected in series between the ground copper plate and the laboratory electrical ground. The leakage current from the discharges produces a voltage drop across the resistor that was monitored and registered with a fast digital insulated oscilloscope (5GSa/s, 0–1000 V, 0.5% + 0.05% voltage range, RTH1004, Rohde & Schwarz, Munich, Germany) equipped with two RT-ZI10 passive voltage probes (500MHz, 1kV, 10:1, R&S ^®^, R&S, Munich, Germany).

Corona appearances in the leakage current is seen as peaks superimposed in the current waveform. Therefore, by using a peak detection algorithm (based on the findpeaks function of Matlab^®^) it is easy to differentiate between corona and no corona conditions.

## 3. Experimental Results

This section details the experimental results obtained by using the setup and instrumentation detailed in Section 2.

### 3.1. Visual Corona Photographs Taken with the Back-Illuminated CMOS Sensor

**Figure 2:**
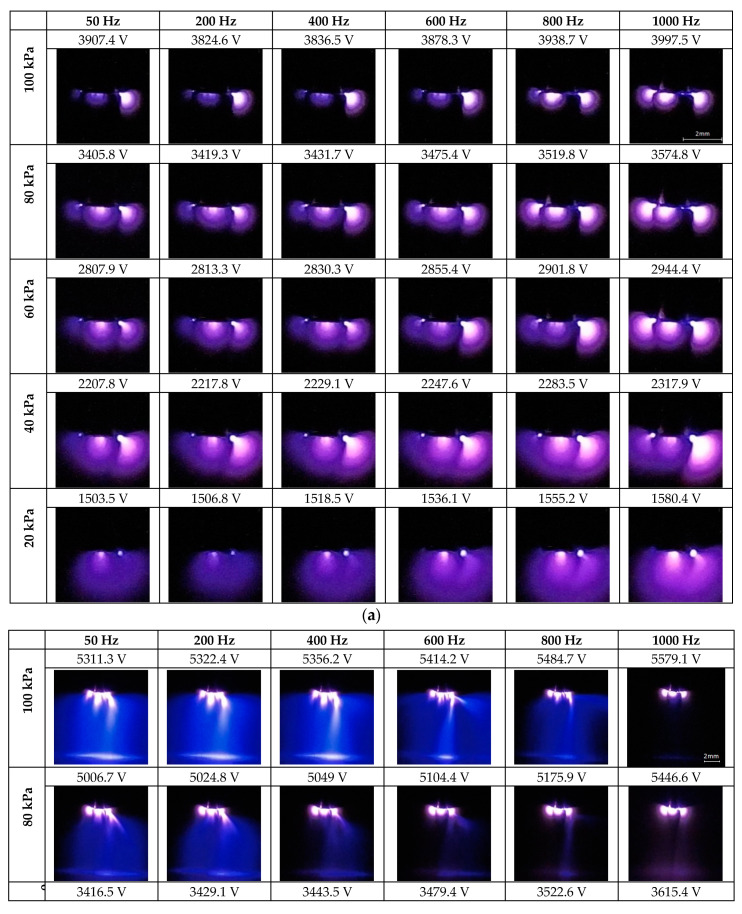

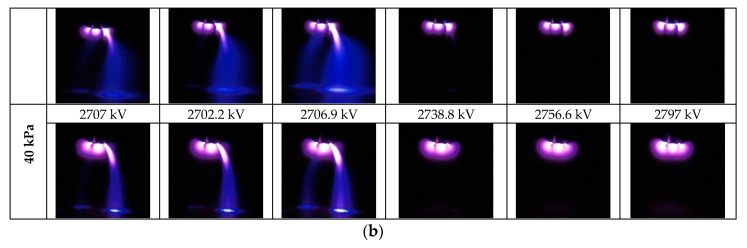
Long-exposure images taken with the back-illuminated CMOS sensor. (**a**) Negative ac corona discharges. (**b**) Positive ac streamer corona discharges.

In order to describe the effects of frequency and pressure on corona discharges, long-exposure photographs (32 s exposition time, RGB mode, ISO 400, manual focus, automatic white balance) were taken using the setup detailed in Section 2. The discharges were performed in the 20–100 kPa range in increments of 20 kPa and for different frequencies (50 Hz, 200 Hz, 400 Hz, 600 Hz, 800 Hz and 1000 Hz). Some of the long-exposure photographs are shown in Figure 2, which show the effects of pressure and frequency on the visual corona discharges. It is noted that the voltage levels corresponding to the photographs in Figure 2 are higher than the CEV values to facilitate a good description of the discharge patterns. It is noted that at low pressure, specifically around 20 kPa, care must be taken when increasing the voltage level, because there is very little difference between the CEV value and the voltage level at which complete breakdown occurs.

Figure 2a shows negative corona discharges before streamers of positive corona appear. The major visual effect is due to the pressure change. At high pressures, corona discharges appear in several spots or “beads” and the active region of ionization is relatively small and well-defined. As pressure decreases, the active region slightly expands and becomes more diffuse, while the number of corona spots reduces. Figure 2a also shows that the supply frequency has very little visual effect on the distribution of the corona discharges.

Figure 2b shows positive corona discharges superimposed with negative discharges, the last ones appearing at lower voltages. According to the images included in this figure, the streamers become more localized and ultimately develop into fewer beams of light (650 µm ± 100 µm in diameter, measured from the images) as pressure decreases. Figure 2b also shows that the density of positive streamers also reduces when the supply frequency increases. It can also be observed that in some cases, for a given pressure, there is a maximum frequency from which streamers are not formed, and a further voltage increase may be followed by electrical breakdown.

### 3.2. Obtained Corona Extinction Voltages (CEV)

This section describes the experimental CEV results attained when analyzing rod-to-plane gap geometry, as described in Figure 1b. To obtain the CEV value, the voltage is progressively increased from 0 kV until identifying corona activity, this point corresponds to the corona inception voltage (CIV). Next, the voltage is increased by about 10% and slowly reduced until the corona effect extinguishes. The last point is where a corona manifest corresponds to the corona extinction voltage (CEV), i.e., the minimum voltage value where corona activity can be found.

**Figure 3:**
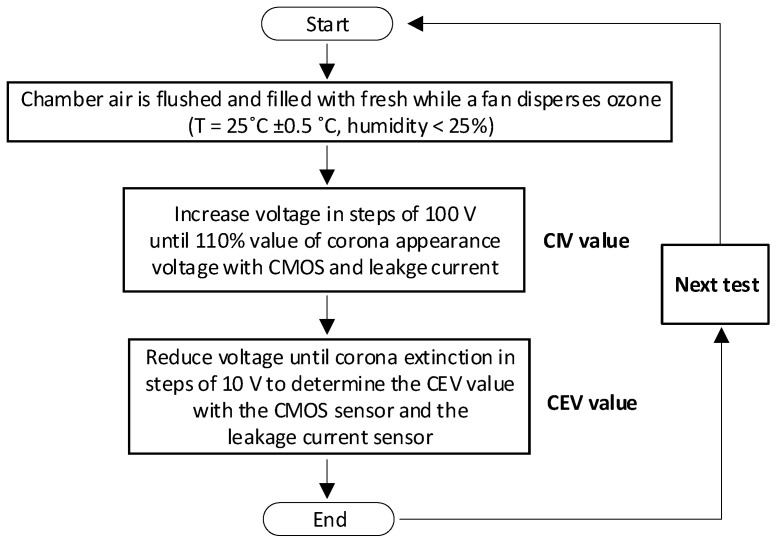
Procedure to determine the value of the corona extinction voltage (CEV).

Figure 3 summarizes the process to determine the CEV value. This process was repeated three times for each measurement, and these values were annotated.

To avoid the CEV value to be influenced by ozone formation, the atmospheric air in the low-pressure chamber was completely replaced in each test.

Table 1 summarizes the tests performed for each condition (pressure range 20–100 kPa, frequency range 50–1000 Hz).

The voltage amplitude was increased with discrete steps of 100 V consisting of a ramp with a standard 1 V/ms rate. To determine the CEV value, the voltage was decreased with steps of 10 V at a rate of −1 V/ms.

Table 2 summarizes the CEV values obtained by using the CMOS imaging sensor according to the experimental setup shown in Figure 1b when analyzing different frequencies (50 Hz, 200 Hz, 400 Hz, 600 Hz, 800 Hz and 1000 Hz) and different pressures (100 kPa, 80 kPa, 60 kPa, 40 kPa and 20 kPa).

**Figure 4:**
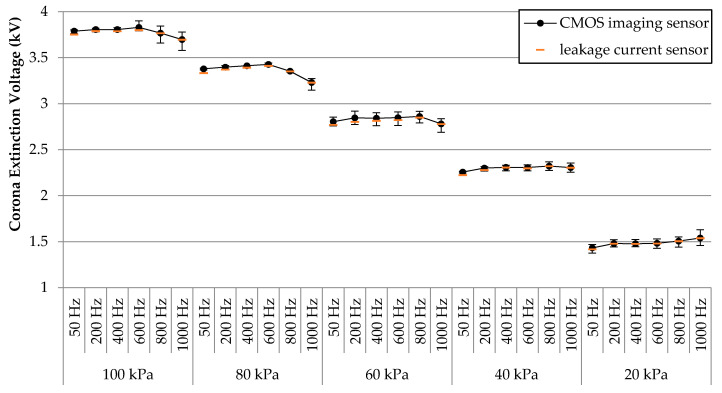
Experimental results of the rod-to-plane electrode geometry. CEV values (kV) versus pressure (kPa) and supply frequency (Hz). Results from the imaging sensor and the leakage current sensor (resistor) were plotted together.

For a better analysis, the results presented in Table 2 are potted in Figure 4.

Results in Figure 4 show that when analyzing the rod-to-plane electrode geometry, the effect of frequency in the range 50–1000 Hz is much less than the effect of pressure in the 100–20 kPa interval in the CEV values. Although high frequencies tend to reduce the CEV values in the 100–60 kPa range, this effect disappears at lower pressures.

**Figure 5:**
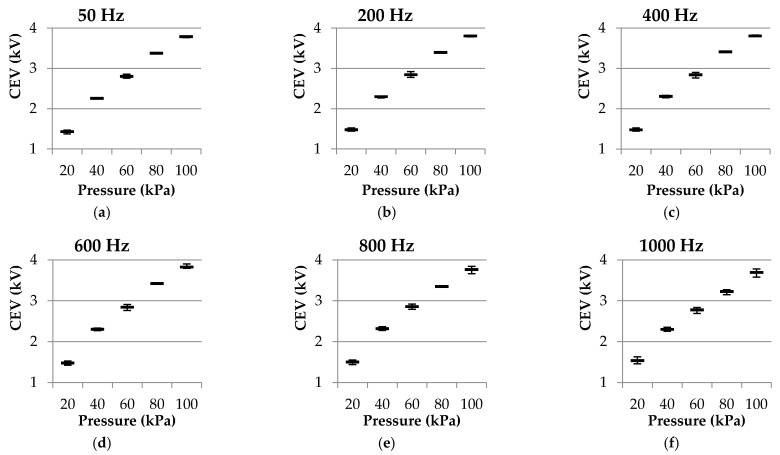
Experimental results of the rod-to-plane electrode geometry obtained with the CMOS imaging sensor. CEV values (kV) dispersion plot of the three measurements at each point versus pressure (kPa). (**a**) 50 Hz; (**b**) 200 Hz; (**c**) 400 Hz; (**d**) 600 Hz; (**e**) 800 Hz; (**f**) and 1000 Hz.

To further analyze the effect of pressure, Figure 5 shows the CEV versus pressure error plots at each analyzed frequency. Such error plots show that the CEV reduces with pressure almost linearly. The parameters of the linear fits are summarized in Table 3.

Results summarized in Table 3 show a quasi-linear relationship of the CEV versus *P* plots measured at different frequencies in the 50–1000 Hz range, according to the high values of the determination coefficient *R*^2^. These results also show similar values of the *CEV*_0_ and *m* parameters for the different frequencies, thus corroborating the low effect of the frequency in the CEV value.

Table 4 compares the CEV values obtained with both sensors.

From the values shown in Table 4 it can be observed that both methods have similar sensitivity, whereas the difference between the results attained with the imaging sensor and those with the leakage current sensor decreases with frequency.

## 4. Discussion

The results presented in Figure 4 clearly show that CEV values are mainly affected by ambient pressure. The results plotted in Figure 4 are in accordance with previous studies analyzing gas discharges for specific supply frequencies [7]. This effect is due to the fact that the mean free path between ion collisions is inversely proportional to air density, and thus, a larger number of successful secondary ionizations are produced at a lower pressure, so that partial discharges can occur at lower voltages than the ones required at atmospheric pressure [39].

Regarding the effect of frequency, Linder and Steele [40] studied the effect of frequency on breakdown, proving that breakdown voltage decreases as the operating frequency increases. There are other studies describing that CIV values usually decrease when increasing the frequency, although this effect reduces at lower pressures [10]. This same effect was observed in the experimental results presented in this paper. However, as can be seen in Figure 4, the CEV values at 20 kPa slightly rise when increasing the frequency up to 1000 Hz.

The formation of a larger number of negative corona spots and brighter negative discharges at atmospheric pressure in contrast to what was observed at low pressure as shown in Figure 2a, can be attributed to the fact that at atmospheric pressure a higher voltage is needed to produce a corona; therefore, more spots are suitable for ionization and more molecules are ionized in the process, thus increasing the brightness of the discharge [41]. The shape change observed in Figure 2a from a localized and defined to a more diffuse and homogenous corona when lowering pressure, may be due to the fact that at a low pressure, the free path of ionization is larger, so that ionized particles can travel further, thus increasing the active area of ionization. This visual effect of pressure on a corona has also been described in previous studies [42].

The results in Figure 2b show that when pressure reduces, the number of streamers also reduces, becoming less diffuse and more localized. This effect was described in [43] using a high-speed photographic camera. In the images presented in this paper, atmospheric-pressure streamers appear as a diffuse bluish glow within the gap due to the 32 s long-exposure effect.

To the best of our knowledge, there is a scarcity of publications analyzing in detail the combined effect of variable frequency and variable pressure on visual corona. However, it has been shown that although frequency has no significant visual effect on negative corona (see Figure 2a), there is a slight effect on the streamers of positive corona (see Figure 2b).

The sensibility to detect corona discharges of the image sensor has been tested and compared with that of the leakage current sensor, obtaining very close results, as shown in Figure 4 and in Table 4, where the percentage differences are calculated, which are very low. A similar comparison was performed in [7] where it was also concluded that the imaging method with a CMOS camera has almost the same sensitivity as other sensitive methods for corona detection.

It is noted that a drawback of the detection method based on the CMOS sensor is that it requires partial darkness to operate. However, partial darkness is often found in aeronautics applications since wires and harnesses are often inside troughs, ducts, or conduits whose interior is usually dark. The authors are aware of this drawback, so they are working in the integration of solar-blind imaging sensors, which can also operate under usual sunlight conditions.

## 5. Conclusions

This paper conducted an experimental study to determine the effect of pressure and frequency on visual corona using a CMOS imaging sensor and by measuring the leakage current, proving that both sensing systems present very similar sensitivity, although the imaging sensor allows locating the points where the electrical discharges occur. The study was conducted by analyzing a rod-to-plane air gap in the 20–100 kPa and 50–1000 Hz intervals, covering most aeronautic applications. The results show that pressure and frequency both have an effect on corona extinction voltage (CEV). CEV increases remarkably with air pressure, but the effect of frequency is lower, causing the CEV to decrease with frequency in the 100–60 kPa pressure range, this effect diminishes with pressure. In addition, a visual description of the effects of pressure and frequency on a corona was performed. The results presented show that the CMOS image sensor has enough sensitivity to be used as a corona detector in low-pressure environments and under a wide range of electrical frequencies. In addition, it was shown that although the difference between the CEV values found with the CMOS imaging sensor and by analyzing the leakage current is very low, this difference tends to reduce at higher frequencies.

## Figures and Tables

**Table 1:** Tests performed (three consecutive repetitions each test).

Pressures	Frequencies
100 kPa	50, 200, 400, 600, 800, 1000 Hz
80 kPa	50, 200, 400, 600, 800, 1000 Hz
60 kPa	50, 200, 400, 600, 800, 1000 Hz
40 kPa	50, 200, 400, 600, 800, 1000 Hz
20 kPa	50, 200, 400, 600, 800, 1000 Hz

**Table 2:** Experimental results corresponding to the rod-to-plane electrode geometry. CEV versus environmental pressure and supply frequency.

Pressure	Test	Sensor	50 Hz	200 Hz	400 Hz	600 Hz	800 Hz	1000 Hz
100 kPa	Test 1	Camera	3761.5	3817.1	3784.0	3792.0	3659.9	3579.1
		Leakage current	3723.2	3788.1	3754.5	3792.0	3659.9	3579.1
	Test 2	Camera	3802.3	3781.0	3826.3	3899.8	3798.5	3732.3
		Leakage current	3774.4	3781.0	3826.3	3835.9	3798.5	3732.3
	Test 3	Camera	3800.7	3819.6	3807.1	3795.8	3843.2	3779.7
		Leakage current	3763.9	3800.5	3786.8	3774.7	3843.2	3779.7
	Average		3788.2	3805.9	3805.8	3829.2	3767.2	3697.0
80 kPa	Test 1	Camera	3382.9	3414.8	3410.6	3441.2	3341.8	3146.4
		Leakage current	3326.5	3377.3	3410.6	3430.5	3341.8	3146.4
	Test 2	Camera	3367.0	3398.1	3412.6	3424.6	3358.8	3272.4
		Leakage current	3348.2	3379.1	3373.0	3404.0	3358.8	3272.4
	Test 3	Camera	3385.4	3377.7	3412.9	3415.1	3358.1	3271.9
		Leakage current	3329.1	3359.3	3393.4	3404.2	3358.1	3271.9
	Average		3378.4	3396.9	3412.1	3427.0	3352.9	3230.2
60 kPa	Test 1	Camera	2757.9	2774.2	2761.2	2763.5	2791.7	2689.5
		Leakage current	2719.8	2736.0	2742.6	2742.2	2781.3	2688.1
	Test 2	Camera	2798.5	2842.9	2861.9	2868.7	2873.7	2813.1
		Leakage current	2778.4	2823.8	2842.3	2837.2	2851.0	2813.1
	Test 3	Camera	2853.7	2918.9	2901.8	2909.8	2916.8	2837.4
		Leakage current	2815.5	2851.7	2862.4	2889.6	2916.8	2837.4
	Average		2803.4	2845.3	2841.7	2847.3	2860.7	2780.0
40 kPa	Test 1	Camera	2255.6	2267.0	2270.0	2270.7	2274.6	2254.3
		Leakage current	2198.5	2248.1	2270.0	2249.5	2274.6	2254.3
	Test 2	Camera	2266.1	2315.4	2330.7	2333.9	2366.7	2354.2
		Leakage current	2227.7	2296.3	2330.7	2333.9	2366.7	2354.2
	Test 3	Camera	2246.4	2316.4	2321.5	2313.3	2322.5	2308.0
		Leakage current	2246.4	2297.3	2321.5	2313.3	2322.5	2308.0
	Average		2256.0	2299.6	2307.4	2306.0	2321.2	2305.5
20 kPa	Test 1	Camera	1468.8	1520.2	1522.6	1529.0	1526.7	1529.8
		Leakage current	1449.3	1520.2	1522.6	1529.0	1526.7	1529.8
	Test 2	Camera	1374.7	1443.1	1444.3	1426.4	1439.3	1458.0
		Leakage current	1355.2	1443.1	1444.3	1426.4	1439.3	1458.0
	Test 3	Camera	1450.2	1474.0	1463.6	1488.5	1552.0	1629.1
		Leakage current	1450.2	1474.0	1463.6	1488.5	1552.0	1629.1
	Average		1431.2	1479.1	1476.8	1481.3	1506.0	1539.0

Electrode diameter = 1.5 mm, tip angle = 90°, electrode tip to plane distance = 8 mm.

**Table 3:** Linear fit parameters *CEV* = *CEV*_0_ + *m·P*, where *CEV*_0_ is the CEV at zero pressure in Volt, *m* is the slope in Volt/kPa and *P* is the pressure in kPa.

Frequency	*CEV*_0_		*m*		*R* ^2^	
	Imaging Sensor	Leakage Current	Imaging Sensor	Leakage Current	Imaging Sensor	Leakage Current
50 Hz	980.5	965.9	29.182	28.908	0.9853	0.9867
200 Hz	1040.1	1031.2	28.755	28.565	0.9845	0.9871
400 Hz	1040.0	1043.4	28.813	28.548	0.9835	0.9839
600 Hz	1033.1	1037.5	29.084	28.765	0.9849	0.9847
800 Hz	1095.4	1093.2	27.771	27.771	0.9820	0.9827
1000 Hz	1138.1	1138.0	26.204	26.204	0.9863	0.9864

*R*^2^ is the coefficient of determination of linear regression, indicating how well data fits.

**Table 4:** Average difference between the CEV values attained with the CMOS image and the leakage current sensors for each frequency.

Frequency	Difference
50 Hz	1.153%
200 Hz	0.695%
400 Hz	0.388%
600 Hz	0.466%
800 Hz	0.078%
1000 Hz	0.003%
